# Metabolic transformations in breast cancer subtypes

**DOI:** 10.1186/2049-3002-2-S1-P48

**Published:** 2014-05-28

**Authors:** Devina Mitra, Stephan Bernhardt, Zita Soons, Gernot Poschet, Ruediger Hell, Rainer Koenig, Ulrike Korf, Stefan Wiemann

**Affiliations:** 1 Division Molecular Genome Analysis, German Cancer Research Center (DKFZ), 69120, Heidelberg, Germany; 2 Maastricht University, 6200, Maastricht, The Netherlands; 3 Centre for Organismal Studies (COS) Heidelberg, University of Heidelberg, 69120, Heidelberg, Germany; 4 Leibniz Institute for Natural Product Research and Infection Biology - Hans Knöll Institute Jena, 07745, Jena, Germany

## Background

Heterogeneity of cancer poses a huge challenge for selecting effective treatment. Gene expression data has helped identify different subtypes also of breast cancer [1] which has led to improved patient stratification and therapeutic strategies. However, treatment of the triple negative (TNBC) subtype, which develops independent of hormone and other receptors, still remains challenging also due to heterogeneity within this subtype [2]. Metabolic transformation in cancer has long been discovered but only recently has this phenomenon gained attention in cancer research. Using the METABRIC dataset [1] of 2000 patients we identified key metabolic pathways that are deregulated in breast cancer and further subdivided these changes into the breast cancer subtypes. Combining bioinformatic modeling with experimental data we aimed to understand the flow of metabolites through pathways and identify potential therapeutic targets.

## Materials and methods

Metabolic genes identified in the METABRIC dataset [1] to be informative on survival were selected to classify patients into good and bad prognosis. We constructed a mathematical model containing reactions of the central carbon and nitrogen metabolism to predict flux distributions in several breast cancer cell lines. For quantification of metabolites in breast cancer cells grown in standard medium we used HPLC.

## Results

Genes in glutamine metabolism and urea cycle were upregulated in patients with bad outcome in the METABRIC dataset [1]. This upregulation was most prominent in the TNBC subtype. Our model predicts that TNBCs have an elevated uptake of glutamine and decompose nitrogen as urea. Using HPLC we identified that glutamine consumption was highest in MDA MB 468 cells and the urea cycle is strongly upregulated in this basal subtype of TNBC.

## Conclusion

We have established a mathematical model of metabolic transformation in breast cancer that predicts the urea cycle to be particularly deregulated in the TNBCs. Using *in vitro* experiments we validated this model and observed that even within TNBC there are metabolic differences. In future we will perform perturbation experiments to investigate if our findings can be exploited for TNBC treatment.
